# Performance and Safety of Hyaluronic Acid-Based Gynaecological Filler for Labia Majora Augmentation: A Randomised Controlled Study

**DOI:** 10.3390/life16071141

**Published:** 2026-07-09

**Authors:** Elena Fasola, Dhouha Dridi, Rebecca Susanna Degliuomini, Giovanni Buzzaccarini, Vittoria Benini, Stefano Salvatore

**Affiliations:** 1 Obstetrics and Gynaecology Unit, IRCCS San Raffaele Scientific Institute, 20132 Milan, Italy; rebecca.degliuomini@gmail.com (R.S.D.); giovanni.buzzaccarini@gmail.com (G.B.); benini.vittoria@hsr.it (V.B.); salvatore.stefano@hsr.it (S.S.); 2 Vita-Salute San Raffaele University, 20132 Milan, Italy; 3 Gyplast Medical Institute, 20129 Milan, Italy; dh.dridi2@gmail.com

**Keywords:** gynaecological filler, vulvar filler, cosmetic gynaecology, regenerative gynaecology, labiaplasty, vaginal rejuvenation, labia majora

## Abstract

**Background:** Labia majora atrophy is a common consequence of aging, hormonal changes, and medical conditions, leading to anatomical changes that affect both aesthetic appearance and sexual function. Hyaluronic acid (HA)-based fillers represent a promising, minimally invasive approach to restoring volume and improving the structural integrity of the labia majora. However, clinical data on their efficacy and safety remain limited. **Objective:** To evaluate the efficacy and safety of a cross-linked hyaluronic acid filler for the treatment of labia majora hypotrophy. **Methods:** This randomised, single-centre, double-arm study included 76 women aged 40–65 years with labia majora hypotrophy, allocated to either a treatment group receiving HA filler injections (*n* = 40) or a control group with no intervention (*n* = 36). Primary endpoints included changes in sexual function, assessed using the Female Sexual Function Index (FSFI) at six months, and anatomical assessment using the Motakef classification as an indirect measure of the labia majora proportion. Secondary endpoints included aesthetic improvement (Global Aesthetic Improvement Scale, GAIS), perceived stress (Perceived Stress Scale, PSS), and patient satisfaction. Safety was assessed through the systematic monitoring of adverse events. Results: At six months, FSFI scores significantly improved in the treatment group compared to the control group (*p* < 0.0001), indicating improved sexual function. GAIS scores demonstrated significantly higher aesthetic improvement in the treated group (*p* < 0.0001). No serious adverse events occurred, and procedure-related pain was minimal (mean NRS: 1.4 ± 1.2). The treatment was well tolerated, with only transient and mild local reactions reported. **Conclusions:** The findings suggest that HA-based gynaecological fillers may represent a safe and potentially effective minimally invasive option for labia majora augmentation, with improvements in sexual function, aesthetic appearance, and patient satisfaction. These results should be considered preliminary and warrant further long-term investigation.

## 1. Introduction

Vulvovaginal atrophy (VVA) is a chronic condition primarily linked to oestrogen deficiency, often emerging during menopause and progressively worsening due to aging and medical comorbidities [1,2]. The external female genitalia, including the mons pubis, labia majora, labia minora, clitoris, and vaginal vestibule, undergo structural modifications over time, leading to symptoms such as dryness, burning, irritation, dyspareunia, and urinary discomfort [1,3,4]. These changes, now referred to as genitourinary syndrome of menopause (GSM), reflect a spectrum of genital, sexual, and urinary symptoms related primarily to mucosal and vaginal tissue alterations rather than isolated external volumetric changes [1].

Labia majora atrophy, often associated with the loss of subcutaneous volume and relative protrusion of the labia minora, is an increasingly reported aesthetic and functional concern among women. Unlike GSM/VVA, which primarily involves mucosal atrophy, labia majora hypotrophy is characterised by external soft tissue volume depletion, with potential implications for friction-related discomfort and psychosocial well-being [5].

The decline in oestrogen receptors in the vaginal and vulvar tissues with age affects cellular proliferation, barrier function, and microbial defence, resulting in the depletion of hyaluronic acid, collagen, and elastin, ultimately contributing to tissue thinning and increased susceptibility to irritation and infection [1,6,7]. As these symptoms are often underreported due to embarrassment or the misconception that they are an inevitable part of aging, many women remain undiagnosed or undertreated despite relevant impacts on quality of life [8].

Treatment options for VVA focus on symptom relief and restoration of the vaginal physiology. Standard therapies include non-hormonal moisturisers and lubricants, as well as vaginal oestrogen therapy. Emerging approaches involve selective oestrogen receptor modulators (SERMs), dehydroepiandrosterone (DHEA) inserts, and energy-based therapies such as laser and radiofrequency, although long-term safety and efficacy data remain limited [8,9].

Alongside these functional approaches, increasing attention has been directed toward the treatment of external vulvar soft tissue aging, particularly labia majora volume loss, which may contribute to altered genital protection and aesthetic concerns. This condition is distinct from GSM/VVA and reflects primarily structural and volumetric changes of the subcutaneous tissue.

Different techniques have been explored for labia majora augmentation, including autologous fat grafting, which offers natural volume restoration but requires surgical harvesting and may be associated with variable resorption over time [10,11,12]. A minimally invasive alternative is the use of hyaluronic acid (HA)-based dermal fillers, which provide immediate volume enhancement with a favourable safety profile, rapid recovery, and reversibility [6,7].

Hyaluronic acid fillers are widely used medical devices for soft tissue augmentation, primarily in aesthetic medicine, and their use in intimate areas has progressively expanded in recent years. Their application in vulvar tissues, including the labia majora, vestibular area, and perineal region, is supported by their ability to improve tissue hydration and elasticity, with potential benefits for patient-reported quality of life [13,14,15,16].

In this context, recent studies have demonstrated that hyaluronic acid (HA) may improve the labia majora volume and tissue quality, with consequent improvements in perceived genital proportions and high patient satisfaction [12]. However, current evidence remains limited and largely based on small clinical series.

Recent studies also suggest that combining hyaluronic acid (HA) injections with adjunctive non-surgical procedures, such as radiofrequency, may enhance tissue quality and patient satisfaction, potentially through the stimulation of collagen remodelling and improved dermal elasticity [9].

Hyaluronic acid is a naturally occurring glycosaminoglycan involved in dermal hydration and extracellular matrix integrity; however, its rapid degradation necessitates cross-linking (e.g., BDDE) to improve its mechanical stability and persistence [17,18]. Preclinical studies have shown that cross-linked hyaluronic acid (CLHA) is well tolerated in soft tissues and may promote local tissue remodelling without a significant inflammatory response. In animal models, the injection of CLHA-based formulations has been associated with increased vascularity and structural changes compatible with tissue regeneration [19], without evidence of distant migration [20].

Cross-linked hyaluronic acid (CLHA) fillers are characterised by variable rheological properties that influence elasticity, cohesivity, and tissue integration, which in turn may affect volumising outcomes in different anatomical sites [21,22,23]. Despite these promising properties, clinical evidence in vulvar applications remains limited.

Although HA fillers are widely used in aesthetic medicine for soft tissue augmentation, their use in the genital region remains largely off-label and should be interpreted within the regulatory framework of each specific product and country.

Despite increasing clinical interest in HA-based approaches, evidence regarding long-term efficacy and safety in vulvar indications remains limited, and standardised protocols are still lacking [6,7,13]. Nevertheless, vulvar rejuvenation, including labia majora augmentation, is increasingly recognised as part of a broader multidisciplinary approach to female genital health and well-being [16,24,25].

Current approaches to vulvar rejuvenation integrate surgical and non-surgical modalities, including laser, radiofrequency, platelet-rich plasma, and hyaluronic acid-based treatments, reflecting a growing interest in individualised management strategies [14,26].

Previous investigations have reported improvements in tissue elasticity, volume restoration, and sexual discomfort following HA injections in the labia majora [7]. However, further controlled studies are needed to standardise techniques and confirm clinical outcomes.

In this context, this study represents a randomised controlled clinical trial designed to evaluate the safety and performance of HA-based fillers for labia majora hypotrophy. The aim was to assess functional and patient-reported outcomes related to tissue restoration and sexual function, with particular attention to minimising the injected volume while maintaining clinical efficacy.

## 2. Material and Methods

### 2.1. Study Design

This study was designed as a randomised controlled trial, conducted at a single centre, with a double-arm, interventional design, aimed at assessing the efficacy and safety of a cross-linked hyaluronic acid (HA)-based filler (Zhoabex G—Rosepharma, Lugano, Switzerland) for the treatment of labia majora (LM) hypotrophy. The study complied with the ethical guidelines of the Declaration of Helsinki and followed the Good Clinical Practice (GCP) principles. The study was registered at ClinicalTrials.gov with number NCT06797167 on 8 April 2022. Both the investigational protocol and the informed consent documents were reviewed and approved by the ethics committee at the study site prior to participant recruitment.

The study was conducted at Vita-Salute San Raffaele University, within the Urogynaecology Section of the Obstetrics and Gynaecology Department, from April 2022 to December 2023.

Eligible participants were females aged 40–65 years, presenting with labia majora hypotrophy requiring volume augmentation. Participants were required to have a body mass index (BMI) below 32 kg/m^2^ and provide written informed consent. Preoperative staging classified the hypotrophy severity as mild, moderate, or severe based on the adipose tissue volume and skin condition.

Exclusion criteria included allergies or contraindications to the device, prior surgery on external genitalia, active infections or inflammatory conditions near the treatment area, a history of vulvar cancer or regional radiation, active herpes simplex/zoster, chronic skin conditions, immune disorders, keloid predisposition, uncontrolled systemic diseases (including endocrine, hepatic, renal, cardiac, pulmonary, or neurological disorders), coagulation disorders or anticoagulant therapy, and the use of substances affecting blood fluidity (e.g., aspirin, NSAIDs, vitamin C). Additional exclusions included pregnancy, breastfeeding, mental incapacity, recent COVID-19 vaccination (within two months), or participation in other investigational studies within the last month.

### 2.2. Study Groups and Randomisation

**Figure 1:**
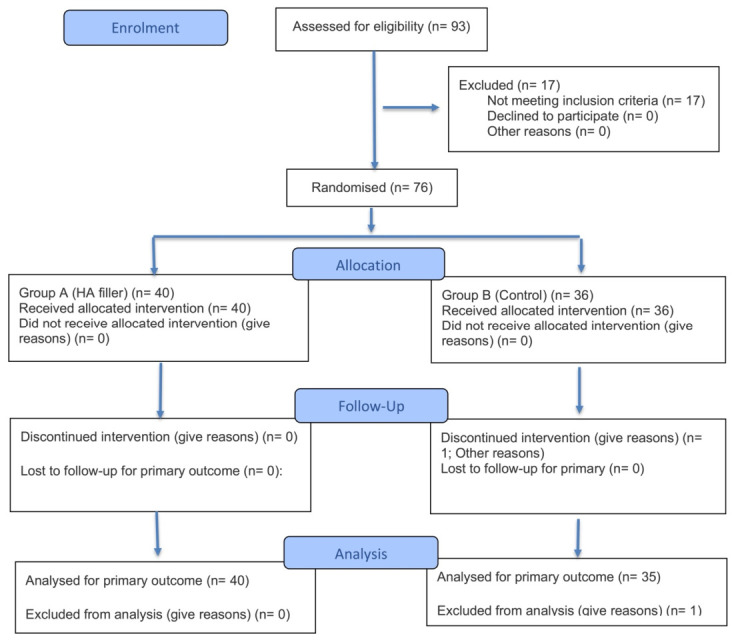
Consort 2025 flow diagram. A total of 93 women were screened for eligibility. Seventeen did not meet the inclusion criteria. Seventy-six participants were randomised and included in the study analyses.

Participants were randomly assigned using a computer-generated block randomisation sequence created with a validated web-based tool (www.randomization.com), without investigator influence. Randomisation was performed at the baseline visit (Visit 0) to ensure balanced group allocation (Figure 1). Participants were assigned to either the treatment group (receiving the HA filler) or the control group (no intervention).

Due to the nature of the intervention, blinding was not feasible; therefore, neither the participants nor the investigators were blinded to group allocation. Allocation concealment was not implemented.

## 3. Materials and Procedure

The study used a cross-linked HA-based dermal filler designed for soft tissue augmentation of the labia majora. The product was supplied in 1.0 mL prefilled syringes containing 25 mg/mL of cross-linked hyaluronic acid with mixed molecular weights (2 MDa and 1 MDa). Excipients included sodium phosphate buffer components, sodium chloride (0.7%), and residual BDDE < 0.1 ppm. The gel was suspended in sterile water for injection.

Procedures were performed under aseptic conditions in an outpatient setting. The treatment area was disinfected prior to injection. A 0.1 mL lidocaine bolus was administered at the cannula entry point.

**Figure 2:**
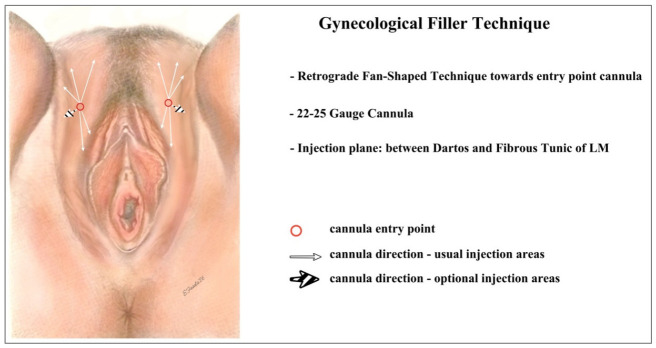
Scheme of MED technique (minimum effective dose technique). *Original drawing by E. Fasola*.

Injection was performed using a 22–25 G cannula in the subcutaneous plane between the superficial and deep adipose tissue, precisely immediately above the fibrous tunic containing the fatty body of the labia majora, using a retrograde fan-shaped technique along the longitudinal axis of the labia majora. The injection strategy followed a standardised* protocol based on the baseline severity (minimum effective dose approach), rather than at operator discretion (Figure 2).

After injection, gentle massage was performed to ensure uniform distribution, followed by topical antibiotic application.

Post-procedure instructions included abstaining from sexual intercourse for 5 days, avoiding pressure or friction for 7 days, and avoiding heat exposure (sauna, pools, intense exercise) for 10 days. A touch-up session was allowed at 1 month only in cases of incomplete correction based on predefined clinical criteria.

Follow-up visits were scheduled at day 7, month 1, month 3, and month 6 to assess efficacy and safety outcomes.

Concomitant medications affecting coagulation, inflammation, or immune responses were not permitted during the study period, except for stable long-term hormonal or contraceptive therapies initiated at least 12 months prior. All medications were recorded.

### 3.1. Outcome Measures

The primary outcome was the improvement in sexual function and genital proportion following HA filler treatment, assessed using the Female Sexual Function Index (FSFI) at 6 months (Visit 4), comparing the treatment and control groups.

Secondary outcomes included sexual function over time, quality of life, symptom severity, pain, aesthetic improvement, and patient satisfaction, using validated instruments.

### 3.2. Endpoints

Primary endpoint:

The primary endpoint was the change in sexual function assessed by the FSFI at 6 months (V4). The FSFI evaluates desire, arousal, lubrication, orgasm, satisfaction, and pain, with scores ranging from 2 to 36, where higher scores indicate better function [27,28].

Secondary endpoints:

1. FSFI changes over time between groups at each visit [29];
2. Quality of life, assessed using the Perceived Stress Scale (PSS) at each visit [30];
3. Labial proportion, assessed using the S. Motakef classification (indirect assessment of labial proportion after augmentation) [31];
4. Aesthetic improvement, assessed using the Global Aesthetic Improvement Scale (GAIS) [32];
5. Symptom severity, assessed using a Visual Analogue Scale (VAS) (Hawker et al., 2011; Williamson & Hoggart, 2005) [33,34];
6. Pain intensity after injection, assessed using the Numeric Rating Scale (NRS) [35];
7. Patient satisfaction, assessed using a 5-point Likert scale [36];
8. Safety, assessed through adverse event monitoring, vital signs, and clinical examination;
9. Expected transient local reactions (oedema, erythema, bruising, tenderness), which were recorded separately from adverse events.

### 3.3. Power Analysis and Statistical Analysis

A sample size of 76 participants was calculated based on the expected differences in the FSFI scores, assuming α = 0.05, 80% power, and a 10% dropout rate.

Statistical analyses were performed using SAS^®^ 9.4. Continuous variables were summarised using descriptive statistics. Normality was assessed using the Kolmogorov–Smirnov test. Appropriate parametric or non-parametric tests were applied depending on the distribution. Repeated-measures analyses were used for longitudinal data. Categorical variables were analysed using frequency distributions.

Both ITT and per-protocol analyses were performed, with ITT as the primary form. The safety analysis included all treated patients. A *p*-value < 0.05 was considered statistically significant.

## 4. Results

A total of 76 patients were enrolled in the study: 40 in the HA filler group and 36 in the control group. All patients in the HA filler group completed the study; in the control group, one patient (2.8%) did not complete the study for non-treatment-related reasons. Looking specifically at the visits, all patients completed V0, V1, and V2, while 75 patients (98.7%) completed V3 and V4 (Table 1).

### 4.1. Demographic and Other Baseline Characteristics

All patients in the HA filler group were Caucasian, while, in the control group, 34 patients (94.4%) were Caucasian, one patient (2.8%) was Black, and one patient (2.8%) was Asian. Twenty patients were smokers (6 in the HA filler group and 14 in the control group), and no patients reported alcohol consumption. Patients in the HA filler group were slightly younger than those in the control group (53.1 ± 6.5 years vs. 54.0 ± 6.9 years, respectively). The mean body weight was 67.0 ± 8.1 kg in the HA filler group and 63.7 ± 6.8 kg in the control group (Table 1).

### 4.2. Medical History

The most common categories of past disorders were as follows:

1. Neoplasms: 8 patients [5 (12.5%) in HA filler group, 3 (8.3%) in control group];
2. Cardiovascular disorders: 11 patients [3 (7.5%) in HA filler group, 8 (22.2%) in control group];
3. Endocrine disorders: 15 patients [4 (10.0%) in HA filler group, 11 (30.6%) in control group];
4. Gastrointestinal disorders: 18 patients [3 (7.5%) in HA filler group, 15 (41.7%) in control group];
5. Previous surgical procedures: 46 patients [21 (52.5%) in HA filler group, 25 (69.4%) in control group] (Table 1).

### 4.3. Trial-Specific Assessment

At baseline (V0), all patients were evaluated using the Motakef classification. In the HA filler group, 35 patients (87.5%) were classified as Class I and 5 patients (12.5%) as Class II. In the control group, 32 patients (88.9%) were classified as Class I and 4 patients (11.1%) as Class II (Table 1).

### 4.4. Compliance with Study Treatment

The HA filler was administered to all patients in the treatment group. At V0, the mean injected volume was 0.5 ± 0.1 mL per side (range 0.1–1.0 mL). At V2, 26 patients (65.0%) required a touch-up procedure, with a mean additional injection volume of 0.5 ± 0.1 mL per side (range 0.3–0.6 mL) (Table 2).

### 4.5. Primary Efficacy Analysis

The primary efficacy endpoint was the performance of the HA filler in correcting labia majora atrophy, reproportioning the labia-minora-to-labia-majora ratio, and improving sexual function, assessed via the Female Sexual Function Index (FSFI).

At V0, the mean FSFI scores were 21.9 ± 4.6 (median 21.0) in the HA filler group and 20.2 ± 7.3 (median 20.3) in the control group; the baseline difference was not statistically significant (*p* = 0.3279).

**Figure 3:**
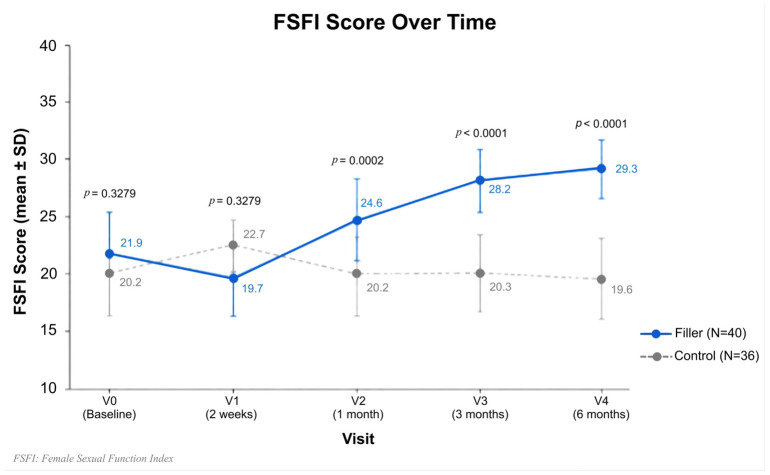
Female Sexual Function Index (FSFI) scores over time in the HA filler and control groups.

At V4 (end of study), the FSFI scores increased to 29.3 ± 3.4 (median 30.1) in the HA filler group and remained stable in the control group (19.6 ± 8.5; median 19.2). The between-group difference at V4 was statistically significant (*p* < 0.0001). The per-protocol analysis confirmed these findings (*p* = 0.0008) (Figure 3, Table 3).

### 4.6. Analysis of Secondary Efficacy Endpoints

**Figure 4:**
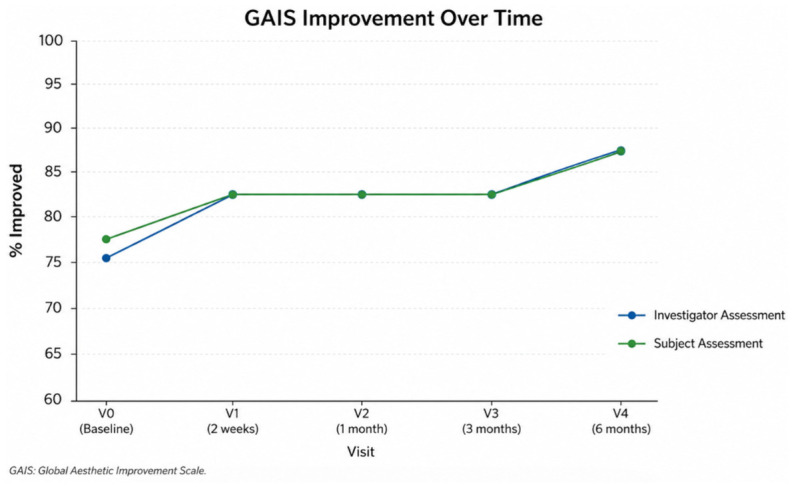
Global Aesthetic Improvement Scale (GAIS)—investigator and subject assessment for patients with outcome “improved” (score 3 GAIS).

**Figure 5:**
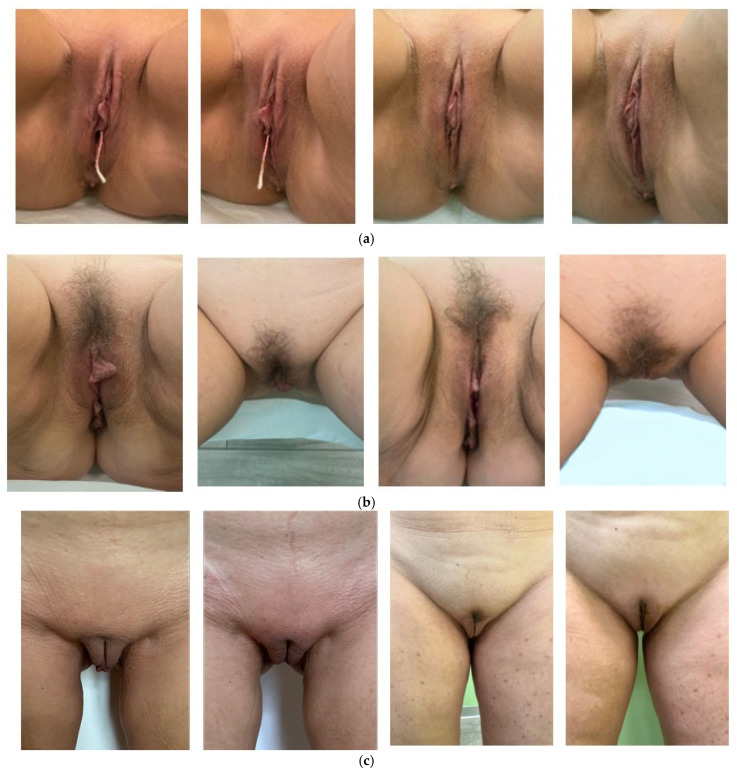

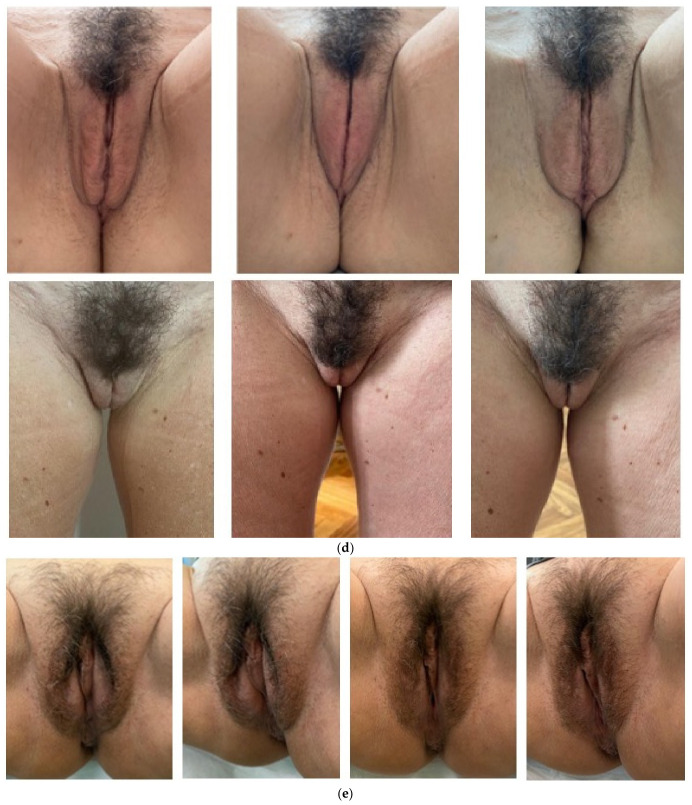
Results after HA gynaecological filler injection by iPhone photo at each visit in 5 perspectives (frontal gynaecological position, ¾ right side and left side in gynaecological position, cranio-caudal direction in gynaecological position, orthostatic position). (**a**) 48-year-old patient—Motakef Class I—before and V4 (after 6 months). Frontal and ¾ sx in gynaecological position. (**b**) 54-year-old patient—Motakef Class II—before and V4 (after 6 months). Frontal and cranio-caudal in gynaecological position. (**c**) This image shows two different cases. The first case refers to a 49-year-old patient—Motakef Class I—before and V4 (after 6 months)—orthostatic position. The second case refers to a 57-year-old patient—Motakef Class I—before and V4 (after 6 months)—orthostatic position. (**d**) 50-year-old patient—Motakef Class I—frontal gynaecological and orthostatic position. (**e**) 61-year-old patient—Motakef Class I—before and V4 (after 6 months)—frontal and ¾ sx in gynaecological position.

1. Sexual function (FSFI): FSFI scores progressively increased in the HA filler group from V0 to V4 (median 21.0 → 30.1), while remaining stable in the control group. Between-group differences were significant from V2 onwards (Table 3).
2. Quality of Life (PSS): PSS scores decreased in the HA filler group from 23.0 (V0) to 19.0 (V4), while remaining relatively stable in the control group (20.0 to 21.0). A statistically significant difference was observed at V4 (mean difference −2.9 ± 5.9; *p* = 0.0440) (Table 3).
3. Labia reshaping (S. Motakef Classification): No statistically significant changes were observed in the Motakef classification in either group across all visits (all *p* > 0.05), consistent with the categorical nature and limited sensitivity of the scale for detecting subtle volumetric changes (Table 3).
4. Vulvar reshaping (GAIS): The Global Aesthetic Improvement Scale (GAIS) was completed independently by both patients and investigators, showing a high level of concordance (Figure 4). In the HA filler group, immediately after treatment, 30 patients (75.0%) were rated as “Improved”, 8 (20.0%) as “Much Improved”, and 1 (2.5%) as “Very Much Improved”. At V4, 35 patients (87.5%) were rated as “Improved” and 5 (12.5%) as “Much Improved”. In the control group, most patients were consistently rated as “No Change” throughout follow-up. Between-group differences were statistically significant at all study visits (*p* < 0.0001) (Table 3, Figure 5).
5. Vulvar atrophy/hypotrophy symptoms (VAS): VAS scores in the HA filler group showed an increasing trend from 5.8 ± 2.9 (V0) to 7.3 ± 1.1 (V4) over the follow-up period (Table 3).
6. Pain intensity (NRS): The mean pain intensity after injection in the HA filler group was 1.4 ± 1.2 (range 1–5) (Table 3).
7. Patient satisfaction (Likert Scale): Patient satisfaction in the HA filler group was high, with a mean Likert score of 3.8 ± 0.7 (range 2–5) at final follow-up (Table 3).
8. Safety: One adverse event (dysuria) was reported in a patient from the control group. The event was classified as mild and non-serious and resolved spontaneously within one day. No treatment-related adverse events or serious adverse events were reported in the HA filler group. Vital signs (systolic blood pressure, diastolic blood pressure, heart rate) and BMI remained stable throughout the study period. Gynaecological examinations revealed occasional findings unrelated to treatment, including vulvar reddening in four patients from the control group and small angiomas of the labia majora in one patient from the HA filler group and two patients from the control group. No device deficiencies were reported during either the initial treatment session or the touch-up procedure (Table 4, Table 5 and Table 6).

## 5. Discussion

In this context, our findings provide preliminary randomised evidence supporting the use of cross-linked HA fillers for the treatment of labia majora hypotrophy. Participants receiving the treatment showed improvements in sexual function and aesthetic outcomes compared with the untreated control group during the 6-month follow-up period. These findings align with the growing interest in non-surgical genital rejuvenation, addressing not only functional symptoms but also the psychosocial burden associated with external genital soft tissue aging and volume loss.

The Female Sexual Function Index (FSFI), a validated instrument for assessing sexual dysfunction, showed significant improvements in the HA-treated group, supporting the potential role of labia majora augmentation in enhancing sexual well-being. This is consistent with previous evidence indicating that the restoration of volume and elasticity in the external genitalia may reduce discomfort, improve lubrication, and increase sexual confidence. Similarly, hyaluronic acid treatment has been associated with improvements in vaginal dryness, dyspareunia, and FSFI scores [37].

Notably, the observed improvements progressed over time, suggesting that HA-based augmentation may provide not only immediate structural enhancement but also sustained functional benefits during the study period. These effects may be partially explained by the rheological and structural properties of the HA filler used, including its viscoelasticity and microstructure, which influence tissue integration and product stability over time [23,24,25,26,27,28,29,30,31].

A key strength of this study is the integration of clinical assessments with patient-reported outcomes. The Global Aesthetic Improvement Scale (GAIS) demonstrated high levels of patient satisfaction, indicating that volume restoration translated into a visible improvement in the appearance of the labia majora. These findings are consistent with previous studies reporting aesthetic improvements and benefits in sexual function and symptom relief [5,38]. In line with the established biocompatibility of HA fillers, our results further support their short-term tolerability in this anatomical region [15,19]. Moreover, comparative studies have shown that HA may improve the labia majora volume and flaccidity, contributing to a more balanced genital appearance [12].

Calcium hydroxyapatite (CaHA) has also been investigated in vulvar rejuvenation due to its biostimulatory properties and ability to induce neocollagenesis. Sequential or combined approaches with HA may represent an interesting future strategy, although comparative clinical studies remain limited.

Histological evidence suggests that combined approaches, such as HA injections with radiofrequency, may enhance tissue remodelling by increasing elastin and collagen deposition in the vulvar skin and vaginal wall [9], potentially contributing to sustained clinical benefits.

Overall, these findings support the hypothesis that labia majora augmentation may contribute to a more youthful and proportionate genital appearance, with potential positive effects on self-esteem and sexual confidence.

While the FSFI and GAIS scores demonstrated significant improvements, the Perceived Stress Scale (PSS) did not show statistically significant differences between groups. This suggests that although HA treatment may improve sexual and aesthetic outcomes, its impact on broader psychological stress is likely multifactorial and may not be directly influenced by anatomical correction alone. Future studies should further explore the relationship between psychological factors and outcomes in genital rejuvenation.

From an anatomical perspective, labia majora augmentation may provide functional benefits beyond aesthetics, particularly by restoring the protective role of the labia majora in reducing friction and microtrauma. Although slight changes in Motakef classification were observed over time, no statistically significant differences were detected between groups. Therefore, the present study cannot confirm anatomical reproportioning as measured by this scale. This finding may reflect the limited sensitivity of the Motakef classification to detect subtle volume changes achieved through minimally invasive augmentation procedures.

No serious adverse events related to the treatment were observed during the study period. Given the limited sample size and follow-up duration, these findings should be interpreted as preliminary evidence supporting short-term tolerability, rather than definitive confirmation of safety. These observations are consistent with the known biocompatibility profile of cross-linked HA fillers and with preclinical evidence demonstrating tissue remodelling without systemic migration [20].

The injection protocol adopted in this study ensured a standardised and reproducible approach, using a fan-shaped retrograde technique with layered infiltration to optimise the volume distribution and minimise complications. The absence of significant pain-related concerns further supports the feasibility of this procedure in outpatient settings. In addition, the repeatability and reversibility of HA-based treatments represent important advantages compared with more invasive surgical options.

Despite the promising results, several limitations should be acknowledged. The study was conducted at a single centre, which may limit the generalisability of the findings to broader and more diverse populations. In addition, long-term durability beyond six months was not evaluated, raising questions regarding optimal retreatment intervals. Evidence regarding durability beyond six months in the vulvar region remains limited. However, studies evaluating cross-linked HA fillers in other anatomical areas have reported the persistence of volumising effects for several months beyond the initial treatment period, depending on the product characteristics and injection technique. Dedicated long-term studies in labia majora augmentation are still needed.

Another important aspect to consider is the potential influence of hormonal status on treatment outcomes. The study population included both perimenopausal and postmenopausal women; however, outcomes were not stratified according to hormonal levels or the use of hormone replacement therapy (HRT). Given the role of oestrogen deficiency in genital tissue aging, future studies should investigate whether hormonal status influences treatment response and whether combined therapeutic approaches may result in improved outcomes.

Additional limitations include the lack of participant and investigator blinding, the use of predominantly patient-reported outcome measures, the absence of objective volumetric assessment techniques, and the relatively small sample size. These factors may have introduced potential sources of bias and should be addressed in future studies.

## 6. Conclusions

This study adds to the limited body of randomised controlled evidence evaluating hyaluronic acid (HA) filler injections for the non-surgical treatment of labia majora hypotrophy. It provides preliminary randomised evidence regarding the clinical performance and short-term tolerability of cross-linked HA-based fillers in gynaecological applications.

The improvements observed in sexual function and aesthetic outcomes highlight the potential of this approach as a minimally invasive option for women experiencing functional and psychosocial distress related to external genital volume loss. No serious treatment-related adverse events were observed during the six-month follow-up period.

Although further research is warranted to optimise treatment protocols, evaluate long-term durability, and confirm these findings in larger populations, the present results support the continued investigation of HA-based augmentation within the broader framework of female genital rejuvenation and sexual health.

## Figures and Tables

**Table 1:** Baseline characteristics of study participants.

Characteristic	HA Filler (N = 40)	Control (N = 36)	Total (N = 76)
Age (years), mean ± SD	53.1 ± 6.5	54.0 ± 6.9	-
Weight (kg), mean ± SD	67.0 ± 8.1	63.7 ± 6.8	-
BMI, mean ± SD	23.2 ± 2.8	22.6 ± 1.4	-
Ethnicity (%)			
- Caucasian	100.0% (40)	94.4% (34)	97.4% (74)
- Black	0.0% (0)	2.8% (1)	1.3% (1)
- Asian	0.0% (0)	2.8% (1)	1.3% (1)
Smoking Status (%)			
- Non-smoker	77.5% (31)	58.3% (21)	68.4% (52)
- Former smoker	7.5% (3)	2.8% (1)	5.3% (4)
- Smoker	15.0% (6)	38.9% (14)	26.3% (20)
Alcohol Consumption (%)	0.0% (0)	0.0% (0)	0.0% (0)
Medical History (%)			
- Neoplasm (including cysts and polyps)	12.5% (5)	8.3% (3)	10.5% (8)
- Cardiovascular disorders	7.5% (3)	22.2% (8)	14.5% (11)
- Endocrine disorders	10.0% (4)	30.6% (11)	19.7% (15)
- Gastrointestinal disorders	7.5% (3)	41.7% (15)	23.7% (18)
- Surgical history	52.5% (21)	69.4% (25)	60.5% (46)
- Reproductive system disorders	10.0% (4)	0.0% (0)	5.3% (4)
Motakef classification (used as an indirect assessment of labia majora hypotrophy based on labia minora protrusion) (%)			
- Class I (0–2 cm)	87.5% (35)	88.9% (32)	88.2% (67)
- Class II (2–4 cm)	12.5% (5)	11.1% (4)	11.8% (9)
- Class III (>4 cm)	0.0% (0)	0.0% (0)	0.0% (0)
Concomitant Medications (%)			
- At least one medication	100.0% (40)	52.8% (19)	77.6% (59)
- Hormonal treatment (stopped >1 year prior)	7.5% (3)	0.0% (0)	3.9% (3)

**Table 2:** Intervention and follow-up data.

Variable	HA Filler (N = 40)	Control (N = 36)
Patients receiving Vagifil injection (%)	100.0% (40)	N/A
Mean volume injected per side (mL)	0.5 ± 0.1	N/A
Local anaesthesia applied (%)	100.0% (40)	N/A
Patients requiring touch-up (%)	65.0% (26/40)	N/A

**Table 3:** Summary of clinical outcomes per visit.

Outcome	Visit	HA Filler (N = 40)	Control (N = 36)	*p*-Value
FSFI Score	V0 (Baseline)	21.9 ± 4.6	20.2 ± 7.3	0.3279
	V1 (2 weeks)	19.7 ± 5.3	22.7 ± 6.2	0.3279
	V2 (1 month)	24.6 ± 4.1	20.2 ± 7.3	0.0002
	V3 (3 months)	28.2 ± 3.7	20.3 ± 7.1	<0.0001
	V4 (6 months)	29.3 ± 3.4	19.6 ± 8.5	<0.0001
GAIS (% Improved) Investigator Assessment	V0 (Baseline)	75.0%	N/A	-
	V1 (2 weeks)	82.5%	0%	<0.0001
	V2 (1 month)	82.5%	0%	<0.0001
	V3 (3 months)	82.5%	0%	<0.0001
	V4 (6 months)	87.5%	0%	<0.0001
VAS (Visual Analog Scale)	V0 (Baseline)	5.8 ± 2.9	-	-
	V1 (2 weeks)	6.9 ± 1.7	-	-
	V2 (1 month)	7.0 ± 1.5	-	-
	V3 (3 months)	7.1 ± 1.3	-	-
	V4 (6 months)	7.3 ± 1.1	-	-
PSS (Quality of Life)	V0 (Baseline)	23.0 ± 5.9	20.0 ± 5.9	0.6474
	V1 (2 weeks)	23.0 ± 5.9	19.5 ± 5.9	0.9668
	V2 (1 month)	22.0 ± 5.9	21.5 ± 5.9	0.5129
	V3 (3 months)	19.5 ± 5.9	22.0 ± 5.9	0.0608
	V4 (6 months)	19.0 ± 5.9	21.0 ± 5.9	0.0440
Post-Procedural Pain Score (NRS)	V0 (Baseline)	1.4 ± 1.2	N/A	-
Patient Satisfaction (Likert Scale)	V4 (6 months)	3.8 ± 0.7	N/A	-
Patients Requiring Touch-up (%)	V2 (1 month)	65.0% (26/40)	N/A	-
Serious Adverse Events	Any Visit	0	0	-
Any Adverse Event (%)	Any Visit	0%	2.8% (1 case, dysuria)	-

**Table 4:** Summary of adverse events.

Treatment Group	HA Filler Group (N = 40)	Control Group (N = 36)
Number of subjects with at least one AE, N (%)	0 (0.0)	1 (16.7)
Number of subjects with at least one related AE, N (%)	0 (0.0)	0 (0.0)
Number of subjects with at least one serious AE, N (%)	0 (0.0)	0 (0.0)
Number of subjects with at least one severe AE, N (%)	0 (0.0)	0 (0.0)
Number of subjects who prematurely terminated the study due to an AE, N (%)	0 (0.0)	0 (0.0)
Number of deaths, N (%)	0 (0.0)	0 (0.0)
Number of AEs, N	0	1
Number of SAEs, N	0	0
Number of related AEs, N	0	0
Number of severe AEs, N	0	0
Number of deaths, N	0	0
*PT dysuria, N (%)	0 (0.0)	1 (100.0)

**Table 5:** Vital signs at each visit.

Visit	Parameter	Statistic	HA Filler (N = 40)	Control Group (N = 36)
V0	SBP (mmHg)	Mean ± SD	122.2 ± 6.2	113.3 ± 8.1
		Min–max	110.0–132.0	100.0–122.0
		Missing	0	0
	DBP (mmHg)	Mean ± SD	77.6 ± 7.3	76.2 ± 4.4
		Min–max	67.0–90.0	68.0–80.0
		Missing	0	0
	Heart rate	Mean ± SD	80.7 ± 11.2	81.0 ± 10.2
		Min–max	65.0–97.0	66.0–97.0
		Missing	0	0
	BMI	Mean ± SD	23.2 ± 2.8	22.6 ± 1.4
		Min–max	19.6–30.5	20.7–24.4
		Missing	0	0
V1	SBP (mmHg)	Mean ± SD	122.2 ± 4.2	115.7 ± 5.9
		Min–max	118.0–130.0	106.0–121.0
		Missing	0	0
	DBP (mmHg)	Mean ± SD	74.0 ± 4.0	73.2 ± 5.2
		Min–max	69.0–79.0	66.0 ± 78.0
		Missing	0	0
	Heart rate	Mean ± SD	77.3 ± 7.0	73.5 ± 4.3
		Min–max	68.0 ± 90.0	69.0 ± 78.0
		Missing	0	0
	BMI	Mean ± SD	23.1 ± 2.9	22.6 ± 1.4
		Min–max	19.6–30.5	20.7–24.4
		Missing	0	0
V2	SBP (mmHg)	Mean ± SD	123.4 ± 4.8	122.7 ± 6.8
		Min–max	115.0–131.0	111.0–129.0
		Missing	0	0
	DBP (mmHg)	Mean ± SD	76.1 ± 5.4	78.3 ± 6.3
		Min–max	66.0–83.0	70.0–85.0
		Missing	0	0
	Heart rate	Mean ± SD	82.7 ± 6.4	83.8 ± 5.6
		Min–max	74.0–93.0	77.0–90.0
		Missing	0	0
	BMI	Mean ± SD	23.2 ± 2.8	22.6 ± 1.4
		Min–max	19.6–30.5	20.7–24.4
		Missing	0	0
V3	SBP (mmHg)	Mean ± SD	117.9 ± 6.1	122.4 ± 8.3
		Min–max	111.0–127.0	111.0–130.0
		Missing	4	1
	DBP (mmHg)	Mean ± SD	77.4 ± 9.6	79.4 ± 7.2
		Min–max	68.0–92.0	70.0–90.0
		Missing	4	1
	Heart rate	Mean ± SD	78.6 ± 8.4	79.4 ± 6.7
		Min–max	70.0–89.0	69.0–87.0
		Missing	4	1
	BMI	Mean ± SD	23.4 ± 3.5	22.6 ± 1.4
		Min–max	19.6–30.5	20.7–24.4
		Missing	4	1
V4	SBP (mmHg)	Mean ± SD	126.0 ± 4.5	122.0 ± 5.2
		Min–max	120.0–130.0	119.0–128.0
		Missing	7	4
	DBP (mmHg)	Mean ± SD	78.0 ± 9.6	77.8 ± 3.1
		Min–max	65.0–88.0	75.0–81.0
		Missing	7	4
	Heart rate	Mean ± SD	83.3 ± 9.6	86.7 ± 4.2
		Min–max	69.0–90.0	82.0–90.0
		Missing	7	4
	BMI	Mean ± SD	24.8 ± 4.1	21.5 ± 0.7
		Min–max	20.7–30.5	20.7–22.1
		Missing	7	4

**Table 6:** Device deficiency.

Timepoint	Device Deficiency	HA Filler Group, N (%)	Control Group, N (%)
V0	Yes	0 (0.0)	0 (0.0)
	No	40 (100.0)	0 (0.0)
	NA	0 (0.0)	36 (100.0)
V2	Yes	0 (0.0)	0 (0.0)
	No	26 (100.0)	0 (0.0)
	NA	0 (0.0)	36 (100.0)

## Data Availability

Restrictions apply to the datasets.
